# Tooth microwear formation rate in *Gasterosteus aculeatus*

**DOI:** 10.1111/jfb.12358

**Published:** 2014-04-28

**Authors:** D C Baines, M A Purnell, P J B Hart

**Affiliations:** * Department of Geology, University of LeicesterLeicester, LE1 7RH, U.K.; ‡ Department of Biology, University of LeicesterLeicester, LE1 7RH, U.K.

**Keywords:** benthic, feeding regime, Gasterosteidae, limnetic, substrate, trophic niche

## Abstract

Tooth microwear feature densities were significantly increased in a population of laboratory-reared three-spined stickleback *Gasterosteus aculeatus* in four days, after they were transferred from a limnetic feeding regime to a benthic feeding regime. These results show that even in aquatic vertebrates with non-occluding teeth, changes in feeding can cause changes in tooth microwear in just a few days, as in mammals.

*In vivo* damage to teeth is apparent when a tooth has been chipped or broken, but there can be more subtle evidence of tooth use. At a microscopic level, the surfaces of teeth can be criss-crossed with scratches and gouges, as well as being pockmarked with pits. This is tooth microwear, evidence of *in vivo* tooth use that can provide information about diet and tooth function during the life of an animal (Walker *et al.*, 1978; Ryan, 1979; Covert & Kay, 1981; Gordon, 1982, 1984, 1986; Teaford, 1991; Solounias & Hayek, 1993). Tooth microwear analysis has become a widely used tool for determining diets in mammals, but only in recent years has it been applied to fishes. This may be because the majority of fishes have simple conical teeth with no occluding surfaces, as it is on these surfaces that in other vertebrates the majority of tooth microwear is formed. In most cases, prey capture in fishes relies on suction (Alexander, 1967; Lauder & Shaffer, 1993) or ram feeding (Liem, 1993), with teeth playing a limited role, such as stopping a prey item escaping from the mouth. Also, teeth in fishes are continually shed and replaced and the functional life of a tooth may be too short to accumulate informative microwear. Previous work, however, demonstrated that tooth microwear data can be recovered from three-spined stickleback *Gasterosteus aculeatus* L. 1758 (Purnell *et al.*, 2006, 2007) and has the potential to be a useful tool for understanding trophic niche in fish populations.

Ecologically, populations of *G. aculeatus* are recognized as occupying trophic niches that range along a spectrum from bottom-feeders (benthic) to plankton-feeders (limnetic), with most freshwater populations falling somewhere along this continuum (Lavin & McPhail, 1985, 1986; Schluter & McPhail, 1992; Caldecutt *et al.*, 2001). Purnell *et al.* (2006) demonstrated that tooth microwear, particularly length and density of features, varies with trophic niche; analysis of microwear can thus be used to determine where along the benthic-limnetic trophic spectrum a population has been feeding. A *proviso* is that to be accurate, analyses must be carried out on fully erupted and fully functioning teeth that have accumulated microwear. This begs an important question: How long does a tooth need to be functioning before it carries a pattern of microwear that reflects trophic niche? Recent work on deer *Capreolus capreolus* (Merceron *et al.*, 2010) and cichlids (Purnell *et al.*, 2012) indicates that microwear data do not always tally with an individual's stomach contents at the time of death because microwear accumulates over a longer period of time than do stomach contents. For individuals, the dietary signal from microwear is therefore less likely to be distorted by opportunistic feeding (Purnell *et al.*, 2012). Turnover in tooth microwear, however, can be rapid, and controlled feeding experiments suggest that, depending on the mechanical properties of food, microwear in mammals may only reflect recent feeding behaviour, rather than diet over a longer period (Teaford & Oyen, 1989). This article addresses the question of how quickly representative tooth microwear accumulates in fishes.

To determine how quickly microwear accumulates on fish teeth, and test the hypothesis that teeth of *G. aculeatus* can rapidly exhibit a significant change in microwear from a limnetic to a benthic pattern, a group of 20 laboratory reared, full sibling individuals (30 mm mean standard length, *L_S_*) was kept under controlled feeding conditions. At the start of the experiment fish were 34 weeks post-hatching and had been kept in a bare tank without substrate (*i.e.* substratum) and fed to satiation with whole defrosted bloodworms (*Chironomus* sp.). Fish were fed in the water column and their teeth had little contact with the walls or floor of the tank. This feeding regime was designed to produce tooth microwear with a low density of microwear features (Purnell *et al.*, 2006) most similar to wild limnetic feeding *G. aculeatus*. Sixteen fish were transferred to a tank with a substrate of clean washed coarse quartz sand (grain size of 500 µm to 2 mm; layer 10 mm thick) and fed to satiation with frozen *Chironomus* sp. daily. Food was introduced to the bottom of the tank by pipette, directly onto the sand to ensure that fish ate off the substrate. This feeding regime was designed to produce tooth microwear patterns like those of benthic feeding *G. aculeatus*, *i.e*. with a high density of features (Purnell *et al.,* 2006).

Prior to the change in feeding conditions (day 0), four randomly selected individuals were killed to record the initial tooth microwear pattern. Subsequently, four fish were sacrificed each day for the next four days (days 1–4). All fish were killed with an overdose of MS-222, and stored in 70% ethanol in individually numbered plastic specimen tubes. Jaws were dissected out following protocols of Purnell *et al.* (2006) with care being taken to ensure that there was no contact between instruments and teeth. During the process of cleaning jaws some of the teeth were accidentally lost from fishes 1/1, 1/2 and 4/3 (first digit relates to day killed and second to individual fish; also see Fig. 2). Seven teeth were lost in total, six from the premaxilla and one from the dentary, leaving a total of 73 for data collection. Dissected jaws were mounted onto standard 12·7 mm scanning electron microscope (SEM) stubs using double-sided carbon tabs, and sputter coated with gold (4 min). Images were acquired from the labial surface of the tooth tip using a Hitachi 3600 SEM (http://www.hitachi-hitec.com) at ×1000 magnification.

Tooth microwear was recorded from the second and third teeth from the symphysis, on the left premaxilla and dentary. Although three-dimensional (3D) methods of dental microwear texture analysis are more robust than 2D approaches, these cannot be applied to teeth of *G. aculeatus* because they are too small, and surfaces too curved for 3D data capture. Images were analysed using the custom software package Microware 4.02 (Ungar, 1995; Ungar *et al.,* 2001); comparative study of operator error and alternative methods of 2D microwear analysis (Grine *et al.*, 2002) advocated use of Microware as a standard approach to quantitative 2D analysis. The collection of microwear data was carried out by a single operator using a Macintosh G5 (http://www.apple.com/mac/) with Windows (http://www.windows.microsoft.com) emulation, with a screen resolution of 1028 × 768 pixels, which resulted in an onscreen magnification of *c.* ×1800. Microwear feature dimensions, orientation and density data were generated as outlined in Purnell *et al.* (2006) and analysed using JMP 9 (http://www.jmp.com). Variables with distributions that differed significantly from normality (Shapiro–Wilks test) were log_10_ transformed prior to analysis.

To test whether microwear, defined by feature major axis length, mean vector length *R*, and feature density, varied over the five days of the experiment (days 0–4), data were analysed by comparing results from the five days in a one-way ANOVA, along with a means comparison of all pairs using a Tukey–Kramer HSD test to reveal any significant differences between days. A least-squares linear regression was also applied to the data and the slope tested for significance (*H*_0_ = slope does not differ from zero). These tests were designed to determine whether any change in microwear had occurred during the course of the experiment.

To determine whether and how quickly during this period of microwear accumulated to the point where it reflected the benthic feeding regime that was imposed on the fish, a one way ANOVA was used to compare experimental data from the five days with data from experimentally controlled limnetic and benthic samples [respectively, limnetic medium substrate (LMS) and benthic course substrate (BCS) data of Purnell *et al.* (2006)]. A Tukey–Kramer procedure was used to test for pairwise differences between each of the five days and the limnetic and benthic samples. Dentary and premaxilla teeth were analysed separately.

One-way ANOVA revealed no significant difference between feature major axis length and mean vector length *R* over the five days of the experiment (Fig. 1 and Table 1). Only feature density varied significantly between days for both upper and lower jaws. For the dentary, the Tukey–Kramer test revealed significant differences in microwear between day 0 and days 2 and 4 but not between days 0 and 3. With the premaxilla, there was also a significant increase in feature density from day 0 but not until day 4. Further investigation of the relationship between microwear values of teeth over the course of the experiment and duration of benthic food consumption revealed significant rank correlations for microwear feature density for both jaws (dentary: *R*_s_ = 0·43 *P* < 0·01; premaxilla *R*_s_ = 0·37 *P* < 0·05) and mean vector length *R* for the dentary (*R*_s_ = 0·33 *P* < 0·05). Regression analysis revealed that, although there was considerable scatter, the relationship between these variables and the day sampled was linear. *r*^2^ Values for regressions were low, but the slopes for dentary *R*, and for feature density on both dentary and premaxilla were significantly different from zero (dentary feature density: slope = 1·66, *F*_1,32_ = 10·89, *P* <0·05, *r*^2^ = 0·23; dentary *R*: slope = −0·04, *F*_1,32_ = 4·96, *P* < 0·05, *r*^2^ = 0·12; log_10_ premaxilla feature density: slope = 0·05, *F*_1,32_ = 5·96, *P* < 0·05, *r*^2^ = 0·17).

**Fig 1:**
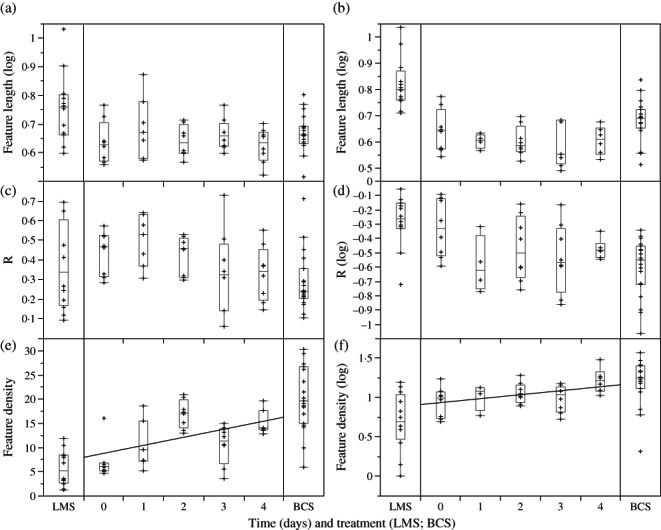
Box plots (1st and 3rd quartiles and median; whiskers are ±1·5 times the interquartile range) showing results of ANOVA of sample means for tooth microwear features (a), (b) feature length, (c), (d) mean vector length (*R*) and (e), (f) feature density of the (a), (c), (e) dentary and (b), (d), (f) premaxilla of laboratory reared *Gasterosteus aculeatus* over the course of the experiment, alongside data from limnetic medium substrate (LMS) and benthic course substrate (BCS) samples of Purnell *et al.* (2006). Where indicated data have been log_10_ transformed. The curves were fitted by: (e) *y* = 8·78 + 1·66*x* and (f) *y* = 0·94 + 0·05*x*.

**Table 1:** Test for tooth differences between days

	*F*	d.f.	*P*
Dentary			
log_10_ *X*	0·9259	4,34	>0·05
*R*	1·8247	4,34	>0·05
Feature density	9·4764	4,34	<0·001
Premaxilla			
*X*	0·8228	4,29	>0·05
*R*	1·5251	4,29	>0·05
log_10_Feature density	2·7345	4,29	<0·05

log_10_, base 10 logarithmic transformation of data that are not normally distributed. *X*, major axis length; *R*, mean vector length.

ANOVA to test whether microwear variables differed from those developed under benthic and limnetic conditions of Purnell *et al.* (2006) revealed that for dentary teeth feature density was significantly greater than limnetic data for the days 2 and 4 samples, confirming the significant shift from conditions at the start of the experiment (Table 2). Also, feature length for day 4 was significantly shorter than for limnetic data. *R* did not differ significantly between experimental groups and limnetic data. Comparison with the benthic sample revealed that feature density data for days 0, 1 and 3 differed significantly from benthic data. There was no significant difference in feature major-axis length between any of the samples and benthic data. *R* differed between groups but in the pairwise comparison only day 1 differed from the benthic data. Premaxilla tooth microwear exhibited a similar pattern. Comparing it with benthic data, no pairwise differences in feature density between treatment day and benthic data were significant. Feature length data differed, but pairwise differences were significant only for day 3. *R* did not significantly differ from benthic data. Comparing premaxilla microwear with the limnetic data, days 2 and 4 differed significantly (higher feature density); all premaxilla samples differed from the limnetic sample in having shorter feature major axis lengths. *R* did not differ.

**Table 2:** Test for differences between daily samples for limnetic medium substrate (LMS; fed limnetically, tanks with medium substrate) fish and benthic course substrate fish (BCS; fed benthically, tanks with course substrate; Purnell *et al.*, 2006)

	*F*	d.f.	*P*
LMS			
Dentary			
log_10_ *X*	3·1621	5,45	<0·05^4^
*R*	1·1917	5,45	>0·05
Feature density	13·2242	5,45	<0·001^2,4^
Premaxilla			
*X*	11·5708	5,40	<0·001^0,1,2,3,4^
*R*	2·8310	5,40	<0·05
log_10_	4·5113	5,40	<0·01^2,4^
Feature density			
BCS			
Dentary			
log_10_ *X*	0·9604	5,52	>0·05
*R*	2·9570	5,52	<0·05^1^
Feature density*	11·1476	5,52	<0·001^0,1,3^
Premaxilla			
log_10_ *X*	3·1324	5,47	<0·05^3^
log_10_ *R*	2·3870	5,47	>0·05
log_10_Feature density	2·5113	5,47	<0·05

*X*, major axis length; *R*, mean vector length.

Superscript numbers refer to daily samples that are grouped separately from LMS and BCS in pairwise tests. log_10_ indicates base 10 logarithmic transformation of data that are not normally distributed.

* A Welch ANOVA *F*-test was used for these data, as the variances of the means were unequal.

The analysis revealed a significant, progressive shift in microwear feature density from limnetic to benthic microwear over the course of the experiment, with feature density reaching significantly different levels to those at the start by day 4 (Figs 1 and 2). Both dentary and premaxilla teeth exhibited comparable patterns (Fig. 1).

**Fig 2:**
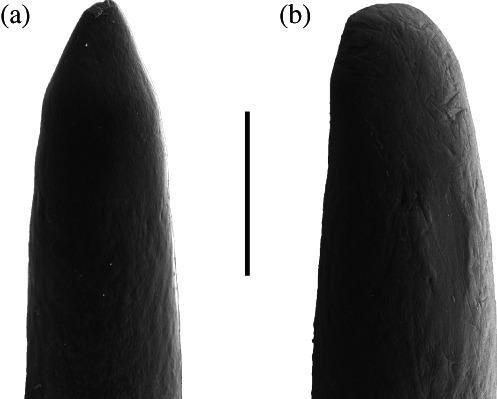
Scanning electron micrographs of representative teeth of *Gasterosteus aculeatus*: (a) tooth from day 0 (image no. 0/1/4) and (b) tooth from day 4 (image no. 4/1/1). The last figure in image number refers to individual teeth. Scale bar = 50 µm.

The change in tooth microwear must be attributable to the change in placement of the food and the introduction of a coarse substrate as the type of food fed to these animals did not change. This alteration of the experimental environment significantly increased the feature density of tooth microwear and demonstrated a rapid shift from the limnetic to benthic range. Under this feeding regime, a newly erupted functioning tooth would acquire a benthic pattern of microwear after 4 days (although the possibility that the shifts in microwear pattern might take place in as little as 2 days cannot be ruled out).

The results do not allow determination of how long it would take for a fish raised in a benthic environment to acquire a limnetic microwear signature after a change in feeding regime. An important factor to consider is that teeth of *G. aculeatus*, unlike the teeth of mammals, are non-occlusal and it is therefore unlikely that microwear will be rapidly polished away but scratches have also been shown to persist over months in the buccal, *i.e.* non-occlusal, tooth surfaces of human subjects (Romero *et al.*, 2012). Consequently, a change from a benthic to limnetic feeding regime is unlikely to result in changes that are as rapid as the limnetic to benthic shift. Teeth from a benthic regime would have relatively high feature densities, and the rate of loss of this pattern and acquisition of microwear representative of a limnetic regime is more likely to be controlled by the rate of loss of teeth. This is because a limnetic feeding pattern, characterized by low feature density, will not polish away and overprint a benthic signature. Preliminary evidence (pers. obs.) suggests that individual teeth in *G. aculeatus* are replaced every few months.

The analysis does not represent a subtle shift in trophic ecology and is likely to be a change of greater magnitude than experienced by fish in the wild. Therefore, the estimate of the rate that microwear can change is likely to be close to the maximum; changes in the wild are likely to be slower. Future investigators are advised to raise fishes in the laboratory and keep them in feeding regimes that produce known microwear patterns as they can temporarily influence subsequent results. Previous studies (Purnell *et al.*, 2012) have suggested that microwear accumulates over a period of several days or more, and analysis of diet based on microwear is therefore less likely to be biased by opportunistic feeding events than analysis of stomach contents (which provide information on what an individual ate in the hours before death). The analysis presented here provides experimental confirmation of this, suggesting that the same will be true for analyses of oral tooth microwear in other species of fishes.
